# Combining ability of banana triploid hybrid progenitors and genomic prediction of cross performance for agro-morphological traits

**DOI:** 10.1093/genetics/iyaf119

**Published:** 2025-06-20

**Authors:** Lucile Toniutti, Simon Rio, Camille Madec, Sébastien Ricci, Chantal Guiougou, Franck Marius, Claude Mina, Jean-Marie Eric Delos, Frédéric Lambert, Jean-Claude Efile, Angélique D’Hont, Guillaume Martin, Jean-Yves Hoarau, Frédéric Salmon

**Affiliations:** AGAP Institut, CIRAD, INRAE, Institut Agro, Université de Montpellier, Montpellier F-34398, France; CIRAD, UMR AGAP Institut, Capesterre-Belle-Eau, Guadeloupe F-97130, France; AGAP Institut, CIRAD, INRAE, Institut Agro, Université de Montpellier, Montpellier F-34398, France; CIRAD, UMR AGAP Institut, Montpellier F-34398, France; AGAP Institut, CIRAD, INRAE, Institut Agro, Université de Montpellier, Montpellier F-34398, France; CIRAD, UMR AGAP Institut, Capesterre-Belle-Eau, Guadeloupe F-97130, France; AGAP Institut, CIRAD, INRAE, Institut Agro, Université de Montpellier, Montpellier F-34398, France; CIRAD, UMR AGAP Institut, Montpellier F-34398, France; AGAP Institut, CIRAD, INRAE, Institut Agro, Université de Montpellier, Montpellier F-34398, France; CIRAD, UMR AGAP Institut, Capesterre-Belle-Eau, Guadeloupe F-97130, France; AGAP Institut, CIRAD, INRAE, Institut Agro, Université de Montpellier, Montpellier F-34398, France; CIRAD, UMR AGAP Institut, Capesterre-Belle-Eau, Guadeloupe F-97130, France; AGAP Institut, CIRAD, INRAE, Institut Agro, Université de Montpellier, Montpellier F-34398, France; CIRAD, UMR AGAP Institut, Capesterre-Belle-Eau, Guadeloupe F-97130, France; AGAP Institut, CIRAD, INRAE, Institut Agro, Université de Montpellier, Montpellier F-34398, France; CIRAD, UMR AGAP Institut, Capesterre-Belle-Eau, Guadeloupe F-97130, France; AGAP Institut, CIRAD, INRAE, Institut Agro, Université de Montpellier, Montpellier F-34398, France; CIRAD, UMR AGAP Institut, Capesterre-Belle-Eau, Guadeloupe F-97130, France; AGAP Institut, CIRAD, INRAE, Institut Agro, Université de Montpellier, Montpellier F-34398, France; CIRAD, UMR AGAP Institut, Capesterre-Belle-Eau, Guadeloupe F-97130, France; AGAP Institut, CIRAD, INRAE, Institut Agro, Université de Montpellier, Montpellier F-34398, France; CIRAD, UMR AGAP Institut, Montpellier F-34398, France; AGAP Institut, CIRAD, INRAE, Institut Agro, Université de Montpellier, Montpellier F-34398, France; CIRAD, UMR AGAP Institut, Capesterre-Belle-Eau, Guadeloupe F-97130, France; AGAP Institut, CIRAD, INRAE, Institut Agro, Université de Montpellier, Montpellier F-34398, France; CIRAD, UMR AGAP Institut, Sainte-Clotilde, La Réunion F-97494, France; AGAP Institut, CIRAD, INRAE, Institut Agro, Université de Montpellier, Montpellier F-34398, France; CIRAD, UMR AGAP Institut, Capesterre-Belle-Eau, Guadeloupe F-97130, France

**Keywords:** *Musa acuminata*, heritability, general combining ability, genomic prediction, breeding

## Abstract

Breeding disease-resistant cultivars that meet commercial criteria is essential to sustain banana production threatened by major diseases. Edible bananas are seedless triploid hybrids that represent end-breeding products. Hence, the crucial step in banana breeding is to improve and combine the parents. Currently, little information is available on parental combining abilities and on the inheritance of major traits to effectively guide banana breeding strategies. In this study, a breeding population of 2,723 triploid individuals resulting from multiparental diploid-tetraploid crosses was characterized during three crop cycles for 23 traits relating to plant and fruit architecture and bunch yield components. The phenotypic variance was partitioned between non-genetic and genetic effects, the latter including the general combining ability of diploid and tetraploid parents, their specific combining ability, and additional variance due to the within-cross genetic variability. Heritability was moderate to high depending on the trait and revealed the predominance of the tetraploid parent's contribution to hybrid performance for most traits. The use of parental genomic information enabled cross-mean performance prediction through genomic relationship matrices of general and specific combining abilities, the latter being partitioned into dominance and across-population epistasis contributions. Predictive abilities often greater than 0.5 were obtained, particularly when the tetraploid parent was observed in other crosses and, for some traits, when neither parent was observed. Information on trait inheritance and genomic prediction of cross-mean performance will help in selecting and combining parents, facilitating the identification of promising hybrids.

## Introduction

Bananas (*Musa* spp.) are a major staple and cash crop in developing countries and the most-eaten fruit in Europe and Northern America (Basic 2015). World production of bananas (both dessert and cooking) was estimated at 135 million tons for a cultivated area of 5.9 million hectares in 135 countries in 2021 (FAO 2024). Over 400 million people rely on bananas to ensure their food and nutritional security in producing countries (Voora *et al*. 2023). Although nearly a thousand different banana varieties are cultivated in the world, 95% of the global production relies on a very limited number of cultivars (Bakry *et al*. 2021). Among them, bananas from the “Cavendish” group, which is represented by a few natural phenotypic somaclonal variants, account for around 57% of global production (Simmonds 1954; Lescot 2023). The reliance on such an extremely narrow genetic base associated with the monocropping system makes the whole banana production vulnerable. These clones propagated through vegetative methods are particularly susceptible to diseases, pests, and current climate change causing reduced production that leads to food insecurity and income loss (Ploetz and Evans 2015). Varietal improvement is therefore a potential lever to sustain world banana production by developing varieties that are resistant to biotic and abiotic stresses and meet production and commercial criteria. These new varieties may also support the transition to less intensive production models (Brown *et al*. 2020).

Cultivated bananas are natural hybrids between species and subspecies of the *Musa* genus originally selected in South East Asia (Simmonds 1962; Perrier *et al*. 2011; Sardos *et al*. 2022; Martin *et al*. 2023). One of the most important selected traits in cultivated bananas has been their ability to produce edible seedless fleshy fruit (Dodds and Simmonds 1948; Brown *et al*. 2020). Cultivated bananas are thus sterile or with a very low level of fertility. One way to achieve complete or almost complete sterility is through the production of triploid individuals (3x), a ploidy level also thought to provide more vigorous plants with larger bunches than diploids (Bakry *et al*. 2021). A common breeding strategy for obtaining progenies of triploid individuals involves crossing a diploid parent (2x) with a tetraploid parent (4x) (Tomekpe *et al*. 2004; Bakry *et al*. 2021).

The breeding strategy developed at CIRAD to obtain triploid hybrids is (i) to select two diploid varieties as parents, (ii) double the number of chromosomes of one of the parents by colchicine treatment to make it tetraploid (2*n* = 4x), and (iii) cross them. Until now, parents have been selected according to their own phenotypic characteristics (*per se* value). For traits with complex genetic architecture involving non-additive effects, parents can be selected on the basis of the performance of their progeny when crossed with other parents. In the latter case, the statistical methodology developed for hybrid breeding, especially in maize (Sprague and Tatum 1942), helps partition the hybrid value into general and specific combining abilities (GCA and SCA) of the parents.

Banana breeding faces several biological and experimental challenges, including low natural fertility levels of breeding germplasm, low seed germination rates, large space requirements for field evaluation, and a long selection crop cycle period. In the CIRAD breeding program, the first phase of field experiments consists of a series of small trials, planted at different times, in which each candidate is not replicated and families are partially aggregated within blocks. Other crops, particularly perennial crops, like intermediate wheatgrass or sugarcane face the same issues of large designs of unreplicated plants along with spatial heterogeneity at an early evaluation stage. Breeders may use various trial layouts (augmented, row–column, incomplete blocks, or p-rep), possibly using repeated controls to estimate error, which enables adjustment for environmental heterogeneity (Hoarau *et al*. 2022). Crain *et al*. (2021) demonstrated that a model accounting for maternal and paternal effects along with the genomic relationship matrix and an autoregressive row–column model of the residual variance was overall the most robust model for their intermediate wheatgrass unreplicated early breeding trials.

Only a limited number of studies have investigated the heritability of banana breeding traits (Tenkouano *et al*. 2012a; Nyine *et al*. 2019; Biabiany *et al*. 2022). In the context of triploid breeding involving a cross between a diploid (2x) and tetraploid obtained by colchicine doubling (4x), no study has been carried out to assess the contribution of each level of parental ploidy to hybrid variance and to quantify within-cross variability. Knowledge of the heritability and genetic variance partitioning for the main banana breeding traits could help increase breeding efficiency by prioritizing phenotyping in the first breeding stages and optimizing the crossing scheme.

Genomic prediction has become a central tool in many animal and plant breeding programs. Before the seminal paper by Meuwissen *et al*. (2001) and Bernardo (1994) proposed the use of markers to infer kinship relationships between parents of the hybrids to be predicted within the framework of GCA and SCA modeling. In the presence of hybrid genotyping, the genetic effects of the marker alleles (additivity, dominance, and epistasis) can be directly modeled to predict the hybrid performance (Vitezica *et al*. 2013, 2017). More recently, the link between the two modeling (GCA and SCA on the one hand, and genetic effects at markers on the other) has been established (González-Diéguez *et al*. 2021), which highlighted the need to account separately for the contribution of dominance and across-population epistasis to the SCA component. In polyploid species, important steps have been made toward taking non-additive effects into account in prediction models (Endelman *et al*. 2018; Endelman 2023) and using those effects to predict cross-mean performance (Endelman 2025). Hybrid genomic prediction has been successfully applied in various crops including maize, sorghum, wheat, rice, barley, sunflower, oilseed rape or sugar beet (see Seye *et al*. 2020 for a recent review). Regarding bananas, the first empirical evidence of genomic prediction in a multi-ploidy population showed promising accuracies for several breeding traits (Nyine *et al*. 2018).

In this study, we evaluated a large triploid multiparental hybrid population for several agro-morphological traits. The first objective was to estimate trait heritability and assess the relative contribution of 2x and 4x GCA effects, SCA effects, and within-cross genetic effects. The second objective was to evaluate the cross-performances over all traits in terms of mean, variance, and progeny size to be generated, in order to maximize the chances of obtaining a progeny with all the desired characteristics, i.e. reaching an ideotype. The last objective was to evaluate to which extent cross-mean performance can be predicted with or without parental genomic information.

## Materials and methods

### Breeding population

Triploid hybrids have been created using parents from the open field *Musa* collection of the Guadeloupe Biological Resources Centre of Tropical Plants (CRB-PT) at CIRAD Neufchâteau station, Capesterre Belle-Eau, Guadeloupe, French West Indies. The parental population contained 38 wild and cultivated diploid accessions representative of known *Musa acuminata* diversity with distinct genomic backgrounds (Table 1; Martin *et al*. 2023). In order to obtain triploid hybrids, crosses between a diploid (2x) and a tetraploid (4x) genitor were made by hand pollination. Tetraploid genitors were derived from chromosome doubling using a colchicine treatment. Seeds were collected from ripe fruits and subjected to embryo rescue as described in Bakry (2008). The ploidy of genitors (2x or 4x) and progenies (3x) was checked by flow cytometry using a standard protocol (Bakry *et al*. 2007). After greenhouse acclimation, the two-month-old seedlings were then transplanted in the field at CIRAD Neufchâteau station, Capesterre Belle-Eau, Guadeloupe, French West Indies (16°05′N, 61°35′W, elevation of 250 m, average rainfall of 3,500 mm, average temperature of 25°C, and soil classified as andosol) from 2011 to 2015. The breeding population consisted of 2,723 triploid hybrids resulting from crosses between the 38 parents and grouped into 116 full-sib families. Variation in fertility among different parental combinations was observed, leading to variation in the number of hybrids per cross from 1 to 188 (Supplementary Table 1).

**Table 1.** Description of genitors used in crosses as diploid, tetraploid, or both.

Parents	Accession number	Classification	Number of crosses	
			Diploid	Tetraploid
Akondro mainty*^a^*	PT-BA-00010	AA cultivar/Mchare subgroup	0	9
Calcutta 4	PT-BA-00051	AA wild/ssp. *burmannicoïdes*	2	0
Chicame*^a^*	PT-BA-00056	AA cultivar/Mchare subgroup	3	16
Cici (Brésil)	PT-BA-00059	AA wild/ssp. *malaccensis*	1	0
Gu Nin Chiao*^c^*	PT-BA-00108	AA cultivar	1	0
Gwanhour	PT-BA-00443	AA cultivar	0	1
Hom	PT-BA-00120	AA cultivar	1	0
IDN 077	PT-BA-00127	AA cultivar	1	2
IDN 110*^c^*	PT-BA-00131	AA cultivar	5	12
IRFA 903*^c^*	IRFA 903	AA cultivar	4	9
Khai Nai On	PT-BA-00148	AA cultivar	2	1
Khi Maeo*^b^*	PT-BA-00150	AA cultivar	8	0
Malaccensis ITC0250	PT-BA-00187	AA wild/ssp. *malaccensis*	1	0
Malaccensis ITC0399	PT-BA-00463	AA wild/ssp. *malaccensis*	1	0
Malaccensis nain	PT-BA-00454	AA wild/ssp. *malaccensis*	9	0
Manang	PT-BA-00190	AA cultivar	8	8
Mlali Mshia Wa Komba (Mayo 11)*^a^*	MAYO-11	AA cultivar/Mchare subgroup	0	1
Mlali Mshia Wa Komba (Mayo 18)*^a^*	MAYO-18	AA cultivar/Mchare subgroup	0	6
Microcarpa	PT-BA-00204	AA wild/ssp. *microcarpa*	1	0
Monyet	PT-BA-00209	AA wild/ssp. *zebrina*	1	0
Ney Poovan	PT-BA-00238	AB cultivar	0	1
Nzumoheli II*^a^*	PT-BA-00251	AA cultivar/Mchare subgroup	1	6
Pa (Musore) N°2	PT-BA-00259	AA wild/ssp. *malaccensis*	1	0
Pa (Patthalong)*^b^*	PT-BA-00261	AA cultivar	9	0
Pahang	PT-BA-00267	AA wild/ssp. *malaccensis*	2	0
Paka	PT-BA-00270	AA cultivar	7	14
Pisang Bangkahulu	PT-BA-00281	AA cultivar	2	0
Pisang Jaran	PT-BA-00292	AA cultivar	7	0
Pisang Lilin	PT-BA-00303	AA cultivar	5	11
Pisang Madu	PT-BA-00304	AA cultivar	11	8
Pisang Pipit	PT-BA-00310	AA cultivar	4	10
Pisang Segun	PT-BA-00319	AA wild/ssp. *malaccensis*	3	0
Sinwobogi	PT-BA-00371	AA cultivar	3	0
THA 052*^b^*	PT-BA-00233	AA cultivar	3	0
Thong Det	PT-BA-00391	AA cultivar	5	0
Tjau Lagada	PT-BA-00393	AA cultivar	1	1
Tuu Gia	PT-BA-00400	AA cultivar	1	0
Zebrina	PT-BA-00433	AA wild/ssp. *zebrina*	2	0

In the manuscript, tetraploid individuals (obtained from chromosome colchicine doubling) are indicated by a concatenation of the diploid name and the letter “T.”

^*a*,*b*,*c*^ Groups of somaclones.

The experimental setup was divided into 48 blocks nested within 12 trials, and hybrids were evaluated only once in the entire experiment. The 12 trials were conducted at the CIRAD Neufchâteau station. Among these, three trials were established in 2011, three in 2012, two in 2013, three in 2014, and one in 2015. Each block contained 56 hybrids and eight control plants: five Cavendish cv 902, one Pisang Ceylan (PT-BA-00286), one Pisang Madu (PT-BA-00304), and one Calcutta 4 (PT-BA-00051). The control plants were selected to represent the diversity of banana varieties, ranging from *M. accuminata* wild types like Calcutta 4 to interspecific hybrids AAB (Pisang Ceylan), and including *M. accuminata* varieties like Cavendish and Pisang Madu. This diversity among the control plants is reflected in their phenotypic characteristics and helps to better adjust for block effects. They were also chosen for their varying resistance to black Sigatoka, ranging from resistant (Calcutta 4) to susceptible (Cavendish), with Pisang Madu and Pisang Ceylan being partially resistant. Among these controls, only Cavendish represents the ideotype of the export dessert banana. Blocks were grouped within trials and each trial contained two to nine blocks, separated from one another by a Cavendish border (Supplementary Fig. 1).

### Genotyping of the parents

Sequencing information for 36 of the 38 parents was gathered from public databases as well as from unpublished data (Supplementary Table 2). Sequencing information was filtered using cutadapt v3.5 (Martin 2011) to remove adapters and quality trim reads. A variant calling was performed by aligning reads against *Musa acuminata* DH-Pahang V4 reference sequence (Belser *et al*. 2021) using the vcfhunter toolbox (Garsmeur *et al*. 2018). The genotyping file was then filtered in order to remove INDEL variants and keep only bi-allelic single nucleotide polymorphisms (SNPs) with no missing data using the vcfFilter.1.0.py tool from the vcfhunter toolbox. The SNPs corresponding to repeated regions of the reference sequence were discarded as in Martin *et al*. (2023), which resulted in a total of 125,245 polymorphic SNPs.

### Phenotyping

The 2,723 hybrids were phenotyped over a period from 2012 to 2017. The constraint of synchronicity between pollen donors and recipient plants, low fertility, and large space requirements for field evaluation meant that production of crosses and hybrid evaluation were staggered over time.

For each plant, 23 quantitative traits related to yield components as well as plant, bunch, and fruit architecture, were measured over three cycles from 2012 to 2017. Crop cycles in the perennial cultivation practice of bananas correspond to successive pseudostems growing from a large underground rhizome, which successively bear a single bunch. Measurements acquired on the same hybrid across successive crop cycles were considered repeated measurements (longitudinal data). Ten traits were measured at flowering, when the last female flower appeared, and 13 traits at harvest, when the first fruit turned yellow (Table 2). Fruit-related traits such as *Fruit Pedicel Length, Fruit Pedicel Diameter, Fruit Weight, Fruit Length, *and* Fruit Grade* were averaged between the internal and external middle fingers of the third hand of the bunch.

**Table 2.** Description of the 23 agronomic traits studied.

Category	Abbreviation	Description	Unit	Measurement period
Plant architecture	PH	*Pseudostem Height* at flowering	cm	Flowering
Plant architecture	PG	*Pseudostem Girth* at 1 m above soil level at flowering	cm	Flowering
Plant architecture	RI	Pseudostem *Robustness Index* (PH/PG)	—	Flowering
Plant architecture	LL	Rank 3 *Leaf* blade *Length*	cm	Flowering
Plant architecture	LW	Rank 3 *Leaf* blade *Width*	cm	Flowering
Plant architecture	LI	*Leaf Index* (LL/LW)	—	Flowering
Plant architecture	NLF	*Number of* standing *Leaves at Flowering*	—	Flowering
Plant architecture	NLH	*Number of* standing *Leaves at Harvesting*	—	Harvest
Bunch architecture	PL	*Peduncle Length*	cm	Harvest
Bunch architecture	PD	*Peduncle Diameter*	cm	Harvest
Bunch architecture	PI	*Peduncle Index* (PL/PD)	—	Harvest
Bunch architecture	BL	*Bunch Length* at full maturity	cm	Harvest
Bunch architecture	BCI	*Bunch Compactness Index* (BL/NH)	—	Harvest
Fruit architecture	FPL	*Fruit Pedicel Length*	mm	Harvest
Fruit architecture	FPD	*Fruit Pedicel Diameter*	mm	Harvest
Fruit architecture	FL	*Fruit Length*	mm	Harvest
Fruit architecture	FG	*Fruit Grade*	mm	Harvest
Yield component	NH	*Number of Hands* on a bunch	—	Flowering
Yield component	NF	*Number of Fruits* on a bunch	—	Flowering
Yield component	NFH	*Number of Fruits per Hand* (NF/NH)	—	Flowering
Yield component	BW	*Bunch Weight* at full maturity	kg	Harvest
Yield component	FW	*Fruit Weight*	g	Harvest
Yield component	DFM	*Days to Fruit Maturity* i.e. Days between flowering and harvesting	Days	Harvest

Text formatted in italic indicates the names used in the manuscript.

#### General 2x–4x genetic model

Several 2x–4x hybrid genetic models were applied in this study that could all be written as follows:


(1) $$Y_{bcuvthr}=\mu +\alpha_{b}+\beta_{c}+D_{u}+T_{v}+(D\times T{)}_{uv}+H_{th}+E_{bcuvthr}$$

where $Y_{bcuvthr}$ is the phenotypic measurement *r* of hybrid *h* of type *t* in block *b* for crop cycle *c*, μ is the intercept, $\alpha_{b}$ is the fixed effect of block b∈{1,…,B},  *B* being the number of combinations of trials and blocks, $\beta_{c}$ is the fixed effect of crop cycle c∈{1,2,3}, $D_{u}$ is the random GCA effect of the 2x parent *u*, $T_{v}$ is the random GCA effect of the 4x parent *v*, $(D\times T{)}_{uv}$ is the random SCA effect between the 2x parent *u* and the 4x parent *v*, $H_{th}$ is the genetic effect of hybrid *h* of type t∈{control,breed}, and $E_{bcuvthr}$ is the error associated with each phenotypic measurement.

Note that the “*r*” index is used only for Cavendish controls, which are the only hybrids repeated within the same block. Also, GCA and SCA terms are only defined for hybrids with type “breed”. For these same hybrids, the genetic effect ($H_{th}$) represents the residual clonal value of the hybrid and is expressed as a genetic deviation from the mean of its cross.

Using matrix notation, the model from Eq. (1) can be written as follows:


(2) $$y=X\theta +Z_{D}g_{D}+Z_{T}g_{T}+Z_{D\times T}g_{D\times T}+Z_{H}g_{H}+e$$

where y is the vector of phenotypes across all three cycles, X is the incidence matrix for fixed effects, θ is the vector of fixed effects, $g_{D}$, $g_{T}$, $g_{D\times T}$, and $g_{H}$ are the vectors of random 2x GCA, 4x GCA, 2x–4x SCA and hybrid effects, respectively, $Z_{D}$, $Z_{T}$, $\;Z_{D\times T}$, and $Z_{H}$ are incidence matrices linking phenotypic observations to their corresponding random genetic effect levels, and e is the vector of errors. All random terms are assumed independent.

For the error term e, the following distribution was assumed: e∼N(0,R⊗I) where R is the covariance matrix between errors of a same plant across different cycles and I is the identity matrix with a size corresponding to the number of individual plants in the experiment. No constraint was assumed on R other than being positive definite, which is commonly referred to as “unstructured” in the linear mixed model literature. This modeling of correlation between errors is induced by the perennial nature of banana experiments in which phenotypic measurements acquired on the same plant across successive crop cycles represent repeated measurements over time (longitudinal data). Model parameters were estimated using ASReml-R v4 (Butler *et al*. 2009).

#### Phenotypic 2x–4x genetic model

This model was first applied considering only phenotypic observations and pedigree information, which was used to partition the genetic variance into GCA, SCA, and within-cross hybrid components and allowed to estimate the genotypic values of hybrids. In a previous study (Toniutti *et al*. 2023), we evaluated three potential statistical models to best assess genetic banana trait performance along with spatial heterogeneity. The model used here was the one evaluated as the most performant that allowed disentangling pedigree effects from block effects.

The distribution of the vectors of random genetic terms from Eq. (2) was the following: $g_{D}∼N(0,\;\sigma_{D}^{2}I_{D})$, $g_{T}∼N(0,\;\sigma_{T}^{2}I_{T})$, $g_{D\times T}∼N(0,\;\sigma_{D\times T}^{2}I_{D\times T})$, and $g_{H}∼N(0,\;\sigma_{H}^{2}I_{H})$ where $\sigma_{D}^{2}$, $\sigma_{T}^{2}$, $\sigma_{D\times T}^{2}$, and $\sigma_{H}^{2}$ correspond to 2x GCA, 4x GCA, 2x–4x SCA and within-cross hybrid variances, respectively, and $I_{D}$, $I_{T}$, $\;I_{D\times T}$, and $\;I_{H}$ are identity matrices with a size corresponding to the number of 2x parents, 4x parents, 2x–4x parent combinations and hybrids, respectively.

Estimates of clonal values (${\hat{Y}}_{uvth}$) were obtained using BLUEs of fixed effects and BLUPs of random effects:


(3) $${\hat{Y}}_{uvth}=\hat{m}+{\hat{D}}_{u}+{\hat{T}}_{v}+(\hat{D\times T}{)}_{uv}+{\hat{H}}_{th}$$

where $\hat{m}$ is the general mean estimated as $\hat{m}=\hat{\mu }+\frac{1}{B}\sum_{b=1}^{B}$  ${\hat{\alpha }}_{b}+\frac{1}{3}\sum_{c=1}^{3}{\hat{\beta }}_{c}$.

Unlike breeding hybrids, controls were highly replicated over the design as they were observed in each block. Therefore, the shrinkage effect induced by calculating the BLUPs of the genetic random terms was very different between breeding hybrids and controls. For analyses that required the comparison of cross-means to the performance of controls, alternative clonal value estimates (${\tilde{Y}}_{uvth}$) were calculated for each hybrid as the average of phenotypic values corrected for block and cycle effects:


(4) $${\tilde{Y}}_{uvth}=\frac{1}{N_{uvth}}\underset{b\equiv (uvth)}{\sum }\;\underset{c\equiv (uvth)}{\sum }\;\underset{r}{\sum }\;(Y_{bcuvthr}-{\hat{\alpha }}_{b}-{\hat{\beta }}_{c})$$

where b≡(uvth) and c≡(uvth) indicate that block *b* and cycle *c* are compatible with hybrid *uvth*, meaning that hybrid *uvth* has phenotypic observations for cycle *c* in block *b*, and $N_{uvth}$ is the total number of observations for the hybrid *uvth* (i.e. three observations corresponding to the three cycles for all hybrids of type “breed” and more for controls).

Similarly, the estimation of cross variances may be underestimated by using hybrid BLUPs that have been shrunk toward the mean. For analyses involving such estimates, we adapted the model by estimating within-cross hybrid variance specifically to each cross with a sufficient number of progenies (i.e. more than 15), and the other crosses were aggregated into a composite cross with a common variance.

#### Genomic 2x–4x genetic model

In addition to the phenotypic model, in which all levels of genetic terms are assumed to be independent, different models were applied that use genomic information (i.e. 125,245 bi-allelic SNPs) to define the covariance structure of GCA and SCA genetic terms.

The GCA terms had the following distribution: $g_{D}∼N(0,\;\sigma_{D}^{2}A_{D})$ and $g_{T}∼N(0,\;\sigma_{T}^{2}A_{T})$ where $A_{D}$ and $A_{T}$ are the 2x and 4x additive genomic relationship matrices, respectively. The additive genomic relationship $(A_{P}{)}_{ij}$ between parent *i* and *j* of ploidy *P* was calculated following Endelman (2023, 2025):


(5) $$(A_{P}{)}_{ij}=\frac{\sum_{m=1}^{M}\;(X_{im}-Pf_{m})(X_{\;jm}-Pf_{m})}{P\sum_{m=1}^{M}\;f_{m}(1-f_{m})}$$

where *M* is the number of SNPs, $X_{im}$ is the allele dosage of the alternative allele of parent *i* at SNP *m*, and $f_{m}$ is the frequency of the alternative allele at SNP *m*. Note that the application of Eq. (5) results in the following relationship: $A_{T}=2\;A_{D}$.

The SCA term results from non-additive effects that include dominance and across-population epistasis (González-Dieguez *et al*. 2021). In this study, we focused on dominance and additive-by-additive across-population epistasis, which gave the following expression for the SCA vector: $g_{D\times T}=d_{D\times T}+i_{D\times T}$ where $d_{D\times T}$ and $i_{D\times T}\;$ are the vectors of contributions of dominance and across-population epistasis to the SCA effect, respectively, with $d_{D\times T}$ and $i_{D\times T}$ assumed independent.

Recently, Endelman (2023, 2025) presented a general expression for the dominant regressor $Q_{im}\;$ of an individual *i* at a marker *m* for any ploidy level: $Q_{im}=\tau_{P}-(X_{im}-\xi_{P}{)}^{2}$ where $\tau_{P}=(P-1)f_{m}(1-f_{m})+\frac{1}{4}$ and $\xi_{P}=(P-1)f_{m}+\frac{1}{2}$. This result was used by Endelman (2025) to propose an estimator of polyploid cross-mean performance that accounts for dominance effects through the expected dominance regressor value of hybrids obtained from a given cross. We adapted these results to the context of 2x–4x crosses and proposed the following distribution: $d_{D\times T}∼N\left(-Fb,\sigma_{D\times T_{\mathrm{dom}}}^{2}D\right)$ where F is the vector of expected genomic inbreeding coefficients of crosses, *b* is a regression coefficient, D is an approximation of the expected dominance genomic relationship matrix between crosses, and $\sigma_{D\times T_{\mathrm{dom}}}^{2}$ corresponds to the variance of dominance contribution to the SCA effect. Both F and D require the calculation of the expected dominant regressor $Q_{uvm}=E(Q_{uvhm}|X_{um},\;X_{vm})\;$ of hybrid *h* between 2x parent *u* and 4x parent *v* at marker *m* conditional on parental genotypes, which notably involves the non-observed allele dosage $X_{uvhm}$ of hybrid *h* (Endelman 2025):


$$Q_{uvm}=\tau_{3}-\xi_{3}^{2}-E(X_{uvhm}^{2}|X_{um},\;X_{vm})+E(X_{uvhm}|X_{um},\;X_{vm})(4f_{m}+1)$$

with:


$$E(X_{uvhm}|X_{um},\;X_{vm})=\frac{1}{2}(X_{um}+X_{vm})$$

$$E(X_{uvhm}^{2}|X_{um},\;X_{vm})=\frac{1}{2}X_{um}+\;\frac{1}{6}(2X_{vm}+X_{vm}^{2})+\frac{1}{2}X_{um}X_{vm}$$

where $X_{um}$ and $X_{vm}$ are the observed parental 2x and 4x allele dosages at marker *m*, respectively. Note the expression of $E(X_{uvhm}^{2}|X_{um},\;X_{vm})\;$ implicitly assumes a polysomic inheritance in the 4x parent (i.e. random bivalent pairing at meiosis).

The expected genomic inbreeding coefficient $(F{)}_{uv}$ of cross *uv* can be calculated as (Endelman 2023):


$$(F{)}_{uv}=\frac{-\sum_{m=1}^{M}\;Q_{uvm}}{\left(\begin{array}{c} P\\ 2 \end{array}\right)2\sum_{m=1}^{M}\;f_{m}(1-f_{m})}$$

and an approximation of the expected dominance genomic relationship $(D{)}_{uv,u^{\prime }v^{\prime }}$ between cross *uv* and $u^{\prime }v^{\prime }$ can be calculated by adapting the formula presented in Endelman (2023):


$$(D{)}_{uv,u^{\prime }v^{\prime }}=\frac{\sum_{m=1}^{M}\;Q_{uvm}Q_{u^{\prime }v^{\prime }m}}{\left(\begin{array}{c} P\\ 2 \end{array}\right)4\sum_{m=1}^{M}\;f_{m}^{2}{\left(1-f_{m}\right)}^{2}}$$

The distribution of the additive-by-additive across-population epistasis contribution to the SCA effect was the following: $i_{D\times T}∼N(0,\;\sigma_{D\times T_{\mathrm{epi}}}^{2}A_{D}⊗A_{T})$ where the covariance structure was calculated as the Kronecker product between 2x and 4x parental additive genomic relationship matrices (Technow *et al*. 2014; González-Dieguez *et al*. 2021), and $\sigma_{D\times T_{\mathrm{epi}}}^{2}$ corresponds to the additive-by-additive across-population epistasis contribution to the SCA variance.

#### Heritability

For each of the 23 studied traits and each of the two statistical models, i.e., with genomic information or without genomic information, the heritability ($H^{2}$) was adapted from Legarra (2016) and Endelman (2023):


$$H^{2}=\frac{E(V_{G})}{E(V_{G})+E(V_{\bar{E}})}$$

where $V_{G}=\frac{1}{N-1}\sum_{k}{\left\|CZ_{k}g_{k}\right\|}^{2}$ corresponds to the sum over all genetic components *k* of the empirical variances applied to each vector $\left(Z_{k}g_{k}\right)$, where $Z_{k}$ is an incidence matrix linking the *N* evaluated hybrids to the elements of the random vector $g_{k}$ (different from incidence matrices in Eq. (2) which have as many rows as observations), $C=I_{N}-\frac{1}{N}J_{N}$ is a projection matrix used to center $\left(Z_{k}g_{k}\right)$, $I_{N}$ is the identity matrix of size *N*, and $J_{N}$ is a square matrix of 1s of size *N*.

The expected value of $V_{G}$ can be computed using the following general formula for quadratic forms (Searle *et al*. 1992):


$$E(V_{G})=\frac{1}{N-1}\sum_{k}[\mathrm{Tr}(Z_{k}^{T}C^{T}CZ_{k}G_{k})+\mu_{k}^{T}Z_{k}^{T}C^{T}CZ_{k}\mu_{k}]$$

where Tr stands for the trace of the matrix (i.e. sum of diagonal elements), $E(g_{k})=\mu_{k}$ and $\mathrm{Var}(g_{k})=G_{k}$. In practice, ReML estimates from $\mu_{k}$ and $G_{k}$ were used.

The following result was obtained for the variance of average errors over cycles $V_{\bar{E}}$ regardless of the model: $E(V_{\bar{E}})=\frac{1}{3^{2}}\sum_{c=1}^{3}$  $\sum_{c^{\prime }=1}^{3}R_{cc^{\prime }}$.

#### Evaluation of cross-mean predictive ability

We investigated the ability to predict cross-mean performances using different models. As a base model, we used the one considering phenotypic and pedigree information only. Different genomic prediction models were applied, in which genomic information was used to define the expectation and covariance structures: a model with genomic information in GCA terms only, models with genomic information in the GCA and SCA terms involving only the contribution of dominance, across-population epistasis, or both, to the SCA effect.

We performed four different types of leave-one-out cross-validation adapted from approaches developed for genomic prediction of maize hybrid performance (Technow *et al*. 2014; Kadam *et al*. 2016; Seye *et al*. 2020), as summarized in Supplementary Fig. 2. The first cross-validation (2x–4x) consisted in discarding each cross one by one provided that both parents were observed in other crosses over the design. The second cross-validation (0–4x) consisted of discarding each cross one by one along with all crosses in which the 2x parent was involved and provided that the 4x parent was observed in other crosses over the design. The third cross-validation (2x–0) was similar to the second one but rather aimed at excluding crosses associated with the 4x parent. Finally, the last cross-validation (0–0) consisted of discarding each cross one by one along with all crosses in which the 2x and 4x parents were involved. Phenotypic prediction without genomic information was not possible in cross-validation scenario 0–0. Note that the number of observations varied slightly between crosses and cross-validation scenarios (Supplementary Fig. 3).

The predictive ability of genomic prediction was evaluated by correlating predicted cross-means to observed cross-means based on clonal values estimates (Eq. 2).

#### Descriptive phenotypic analyses

Genotypic correlation between all pairs of traits was calculated as Pearson correlation coefficients using estimated clonal values obtained from Eq. (3). All pairwise correlations were tested and sequential Bonferroni correction was applied at a significant level of 5% to correct for multiple testing (Rice 1990).

To assess cross-mean performance, we considered data from the 54 crosses with more than 15 progenies to allow for accurate cross-mean estimates. These 54 families represented 2,434 hybrids or 89% of the initial hybrids. A principal component analysis (PCA) was carried out on the cross-mean performance of each of the 54 biparental families (and the four controls) for all 23 traits obtained from Eq. (3), using the FactomineR R-package.

#### Progeny size per cross

In addition to investigating cross-mean performance using PCA, we investigated the variability generated by each cross for the 20 traits for which it was possible to determine an ideotype. The remaining three traits: *Leaf Length, Leaf width*, and *Leaf Index* can be considered as descriptive traits and are not directly selected in the breeding program. From the mean $\mu_{uv}$ and variance $\sigma_{uv}^{2}$ of each cross *uv*, we calculated the minimum progeny size ($N_{\min}$) that must be generated to get at least one offspring, with probability p=0.9, whose performance is above (or below) a threshold ($y_{\mathrm{ref}}$) if the ideotype is a high (or low) value for the trait. The threshold considered was the clonal value estimate of the Cavendish control included in the experiment (Eq. 2). Assuming a normal distribution for hybrid values $Y_{uvh}∼N(\mu_{uv},\;\sigma_{uv}^{2})$, $N_{\min}$ can be calculated from the probability *p* of getting at least one offspring above the threshold, which can be more conveniently expressed as:


$$p=1-P(Y_{uv1}<y_{\mathrm{ref}}\cap Y_{uv2}<y_{\mathrm{ref}}\cap \ldots \cap Y_{uvN_{\min}}<y_{\mathrm{ref}})$$

Assuming independence between all hybrids from the cross, one obtains:


$$p=1-P(Y_{uvh}<y_{\mathrm{ref}}{)}^{N_{\min}}$$

which can be rearranged to isolate $N_{\min}$:


$$N_{\min}=\frac{log(1-p)}{log\;P(Y_{uvh}<y_{\mathrm{ref}})}$$

where $P(Y_{uvh}<y_{\mathrm{ref}})$ is the value of the cumulative distribution function of the normal distribution for $y_{\mathrm{ref}}$. In practice, cross-means ${\hat{\mu }}_{uv}$ were estimated from clonal value estimates (Eq. 4) and cross variances ${\hat{\sigma }}_{uv}^{2}$ were estimated directly from the model specifying a hybrid variance specifically to each cross. Like for the PCA, only the 54 biparental families with more than 15 progenies were considered to allow for accurate variance estimates.

## Results

### Partitioning of heritability in GCA and SCA effects

**Fig. 1.**
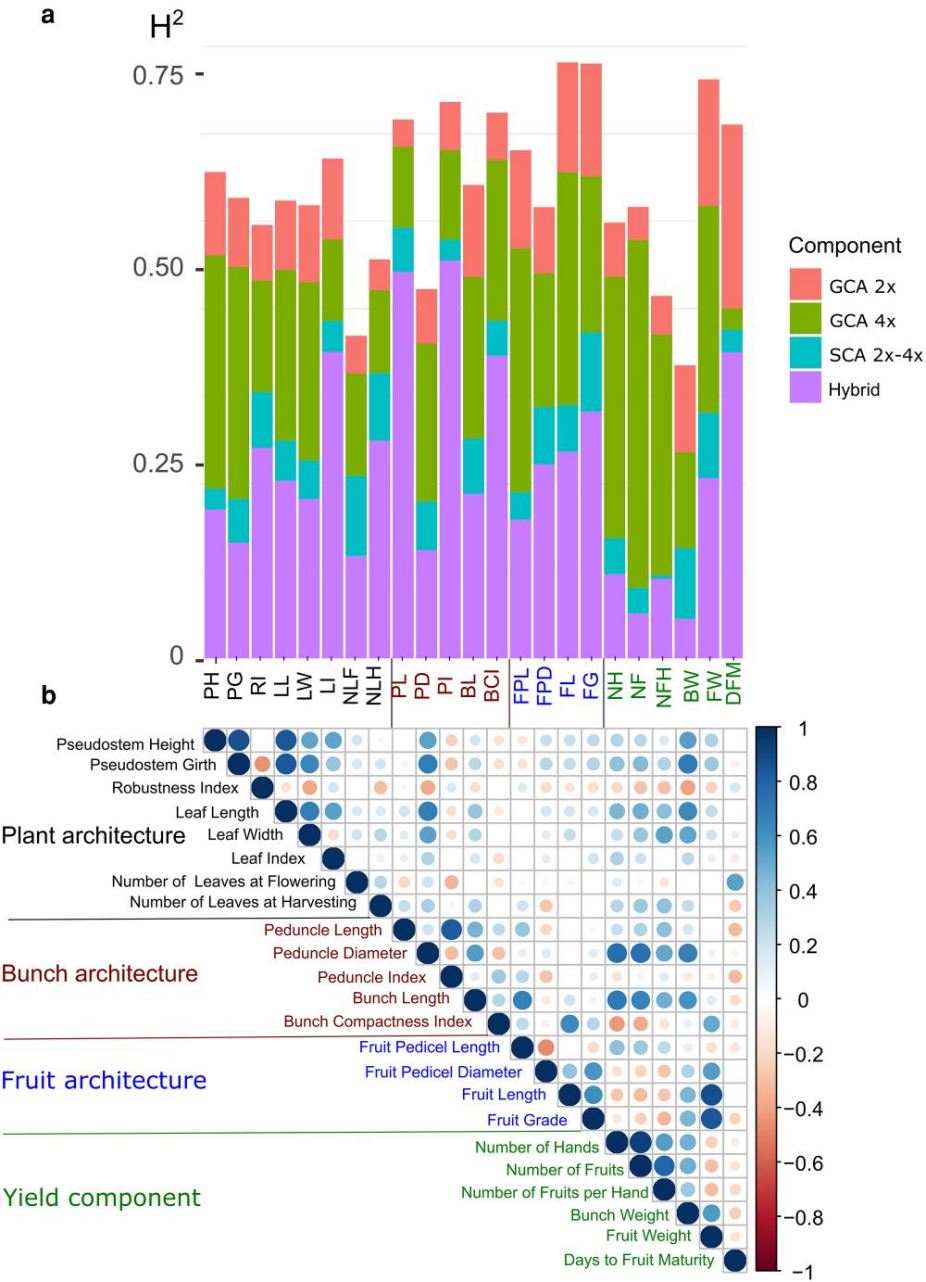
Broad-sense heritability estimates without genomic information (a) and genotypic correlations between 23 traits (b). a) Barplot displaying the partitioning of the heritability into the following components: general combining ability of the diploid (GCA 2x) and tetraploid (GCA 4x) parents, specific combining ability of the parents (SCA 2x–4x), and the within-cross hybrid variability (Hybrid). b) Genotypic correlations between phenotypic traits. Only significant correlations (*P* < 0.05) after Bonferroni correction are shown.

The 23 studied traits showed substantial phenotypic variation with moderate to high estimated heritability ranging from 0.419 for *Bunch Weight* to 0.852 for *Fruit Length* (Fig. 1a; Supplementary Table 3). The cycle effect and block effect were significant for all traits. The model applied, referred to as the phenotypic 2x–4x model, partitions the heritability into the following components: GCA of the 2x and 4x parents, SCA of the parental combination, and a within-cross hybrid variability (Supplementary Table 3). The significance of each random term was evaluated using the likelihood ratio test applied to nested models (Supplementary Table 4). For all traits, except *Days to Fruit Maturity* and *Leaf Index*, the contribution of the 4x GCA to the heritability was larger than the contribution of the 2x GCA (Fig. 1a; Supplementary Table 3; Supplementary Table 4). This superiority of the first variance component over the second one even exceeded a ratio of 3 to 1 for six traits (*Bunch Compactness Index*, *Number of Fruits*, *Number of Fruits per Hand*, *Number of Hands*, *Peduncle Length,* and *Pseudostem Girth*). The only trait showing inverse superiority between parental contributions was *Days to Fruit Maturity*, with a contribution of 0.26 and 0.03 to the heritability for the 2x and 4x GCAs, respectively. *Leaf Index* showed an equal contribution between 2x GCA and 4x GCA. When comparing a model with a common variance for 2x and 4x GCA terms to a model with specific variances, the likelihood ratio test was significant for only five traits: *Days to Fruit Maturity, Number of Fruits, Number of Fruits per Hand, Number of Hands*, and *Pseudostem Girth*.

The SCA contribution to overall genetic variance was low but proved to be significant for all traits, except for *Number of Fruits per Hand* (Supplementary Table 3; Supplementary Table 4). The within-cross hybrid variability contribution to the heritability was highly dependent on the trait, ranging from 0.057 to 0.57 and was the main component for nine traits, namely *Robustness Index, Leaf Index, Number of leaves at Harvesting, Peduncle Length, Peduncle Index, Bunch Compactness Index, Fruit Pedicel diameter, Fruit Grade,* and *Days to Fruit Maturity*.

### Genotypic correlations among agronomic traits

The genotypic correlations between traits were estimated based on the hybrid value estimates obtained from Eq. 2 (Supplementary Table 5). Over the 253 comparisons, 222 pairs showed a correlation coefficient significantly different from zero. Among them, 57 coefficients were higher than 0.4 in absolute value (Fig. 1b). Significant correlations were observed within trait categories. Among traits related to plant architecture, a group of traits (i.e. *Pseudostem Height*, *Plant Girth*, *Leaf Length*, *Leaf Width*, and *Leaf Index*) were positively correlated with each other with correlations higher than 0.50. Regarding bunch architecture traits, the most noticeable correlations were obtained between *Peduncle Length*, *Peduncle Diameter,* and *Bunch Length* with values ranging from 0.21 to 0.55. Regarding fruit architecture traits, *Fruit Pedicel Diameter*, *Fruit Length* and *Fruit Grade* had significant positive correlations ranging from 0.39 to 0.62 whereas *Fruit Pedicel Length* was negatively correlated to *Fruit Pedicel Diameter* and *Fruit Grade* with a correlation of −0.47 and −0.17, respectively. High positive correlations were observed among yield traits: *Number of Hands*, *Number of Fruit,* and *Bunch Weight*, with values superior to 0.48. However, *Fruit Weight* was positively correlated to *Bunch Weight* (*r* = 0.56) but negatively correlated to *Number of Fruits* (*r* = −0.31) and *Number of Hands* (*r* = −0.24). Significant correlations were also observed between trait categories. Plant architecture traits were positively correlated with *Peduncle Diameter* related to bunch architecture (*r* > 0.54) and with yield component traits such as *Bunch Weight* (*r* > 0.53). Bunch architecture traits, especially *Peduncle Diameter* and *Bunch Length*, had a strong positive correlation with yield component traits such as *Number of Hands*, *Number of Fruits,* and *Bunch Weight* (*r* > 0.6). Fruit architecture traits especially *Fruit Pedicel Diameter*, *Fruit Length,* and *Fruit Grade* were strongly correlated to yield components such as *Fruit Weight* and *Bunch Weight* with correlations ranging from 0.30 to 0.88. *Number of Leaves at Harvesting* was positively correlated to yield component traits (*r* > 0.25).

### Comparative cross-performance

**Fig. 2.**
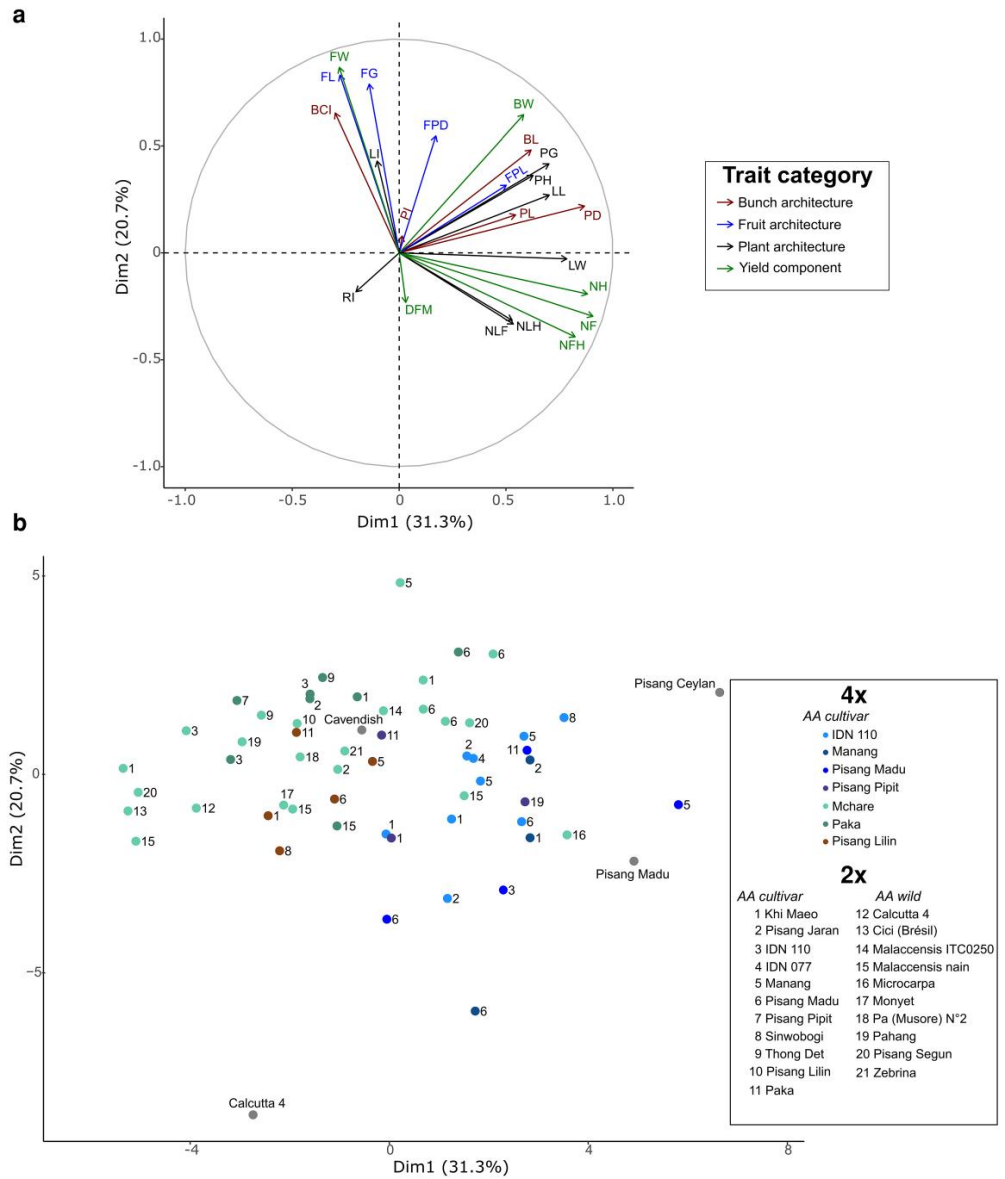
Principal component analysis of cross-mean performance for agro-morphological traits. Trait correlation plots are presented for axes 1 and 2 (a). Cross-mean performance is plotted in the phenotypic space for axes 1 and 2 (b). In panel (a), traits are colored by trait category. In panel (b), dots are numbered according to the diploid parent (2x) and colored according to the tetraploid parent (4x). Cross-mean performance was estimated using Eq. 3 for crosses with a progeny size of at least 15. Controls (grey dots) include Cavendish, Pisang Madu, Pisang Ceylan, and Calcutta 4. Total variance explained by each PC is listed in the axes.

The mean performance of 54 intraspecific crosses along with the performance of the four controls was analyzed using a PCA over the 23 evaluated traits. The first two principal components explained 31.3 and 20.7% of the total variance, respectively (Fig. 2). The 48% remaining variation was spread among components 3 to 23. Traits related to plant architecture, bunch architecture, and yield component contributed to the first component, in particular *Number of Fruits, Number of Hands*, *Peduncle Diameter*, *Leaf Width,* and *Leaf Length* with correlations to the first component equal to 0.91, 0.88, 0.87, 0.79, and 0.70, respectively (Fig. 2a). Traits related to fruit architecture and *Fruit Weight* contributed to the second component, in particular *Fruit Length* and *Fruit Grade* with correlations to the second component equal to 0.87, 0.83 and 0.79, respectively (Fig. 2a). We observed that families were well spread out over the two components of the PCA (Fig. 2b). Only families with small fruits and low yield on average were underrepresented (bottom left of the PCA). Many crosses gathered around Cavendish, especially those including Mchare and Paka as tetraploid parents. Very few crosses narrowed Calcutta 4 wild accession or Pisang Ceylan AAB triploid cultivar on both two first axes. The absence of hybrids outperforming Pisang Ceylan on both PC1 and PC2 can be explained by the differing breeding goals and genetic backgrounds of interspecific vs intraspecific hybrids. Crosses with the same tetraploid parents tended to cluster together while crosses with the same diploid parents tended to be more scattered.

**Fig. 3.**
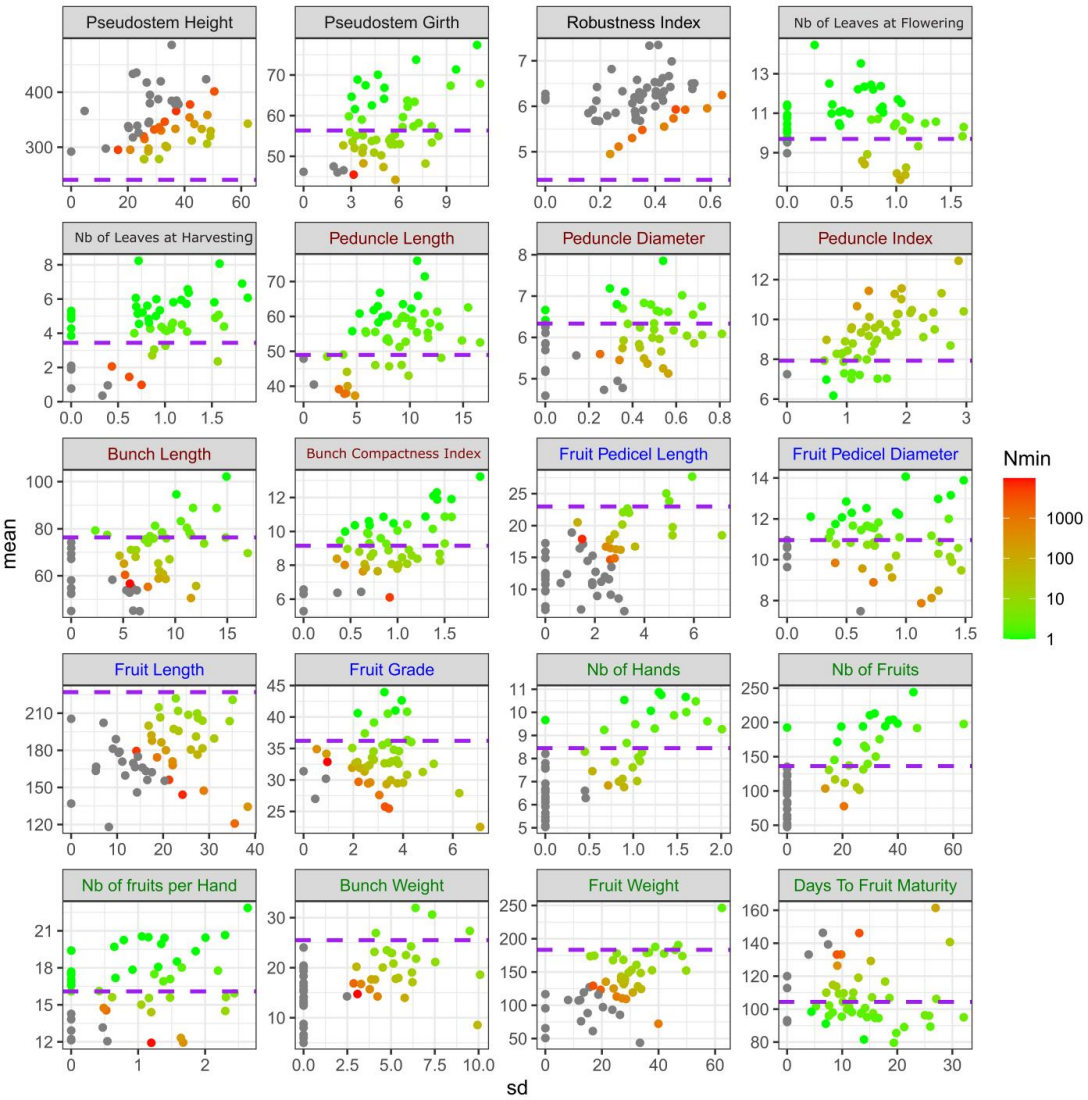
Minimum progeny size required to outperform Cavendish for 20 agronomic traits. For each trait, the cross-mean is plotted against the cross-standard deviation (SD). The purple dashed line shows the Cavendish value. Trait names are colored according to their category. Each point is colored according to the progeny size necessary to obtain at least one offspring with better performance than Cavendish, with a probability of 0.9. For most traits, the selection objective is to have a higher performance than Cavendish, except for *Pseudostem Height*, *Robustness Index*, *Peduncle Index*, *Peduncle Index*, and *Days to Fruit Maturity* for which the objective is a lower performance than Cavendish.

The cross-performances were evaluated over 20 traits in terms of mean, variance, and progeny size to be generated to have an offspring with a better performance than Cavendish (Fig. 3; Supplementary Table 6). Three traits (*Leaf Length*, *Leaf Width,* and *Leaf Index*) were not used because they are descriptive traits for which it is not clear whether a higher or lower value than Cavendish is expected.

Large differences in average cross performance were observed between crosses for key agro-morphological traits. For instance, the mean *Fruit Grade* ranged from 22 to 44 mm and the mean *Fruit Length* ranged from 118 to 222 mm. Large differences could also be observed regarding the standard deviation of the cross-performance according to the trait. For instance, the standard deviation of *Fruit Grade* ranged from 0.001 to 7.1 mm and the standard deviation of *Fruit Length* ranged from 0.09 to 38 mm. For some cross-trait combinations, null estimates of variance were obtained, which likely indicates that the actual variance is very low but not truly null.

The mean and the standard deviation of the cross performance were strongly correlated for some traits (e.g. a correlation of 0.77 for *Number of Hands*) but not for others (e.g. a correlation of 0.01 for *Fruit Pedicel Diameter*).

**Fig. 4.**
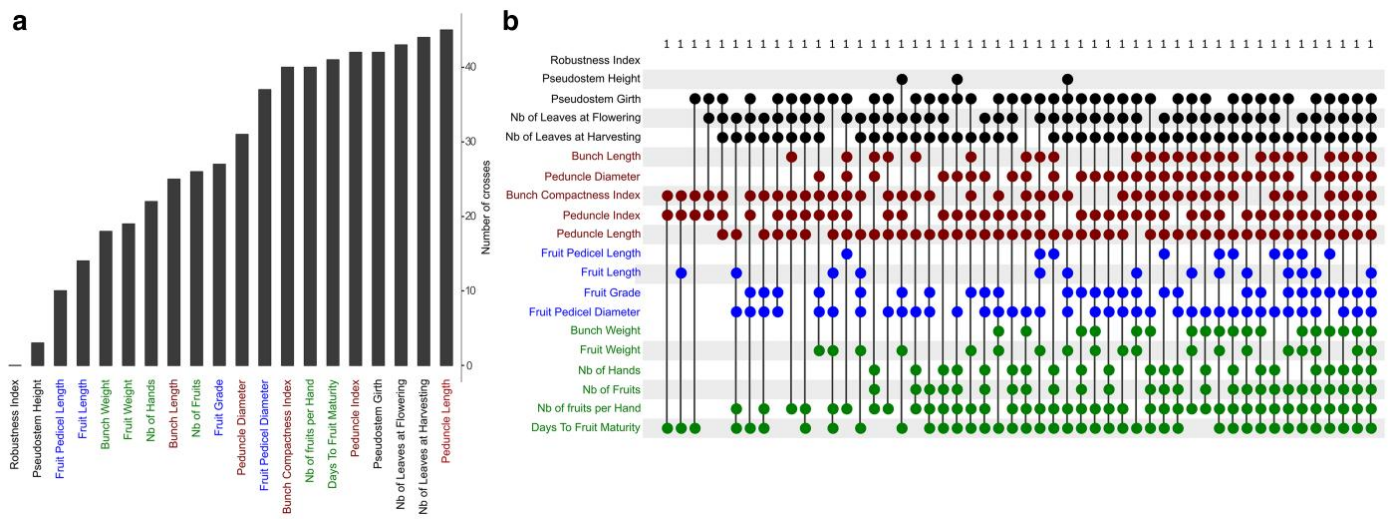
Number of crosses producing individuals outperforming Cavendish. A cross outperforming Cavendish for a trait was defined as a cross for which, with a progeny size of 30, at least one offspring with a better performance than Cavendish should be obtained with probability of 0.9. Trait names are colored according to their category. a) Percentage of crosses outperforming Cavendish for each trait. b) Upset plot showing the number of crosses outperforming Cavendish in each combination of traits. The number of crosses is written on the top of the figure.

For some traits, most crosses theoretically allow a hybrid with better performance than Cavendish with 30 seeds generated (Fig. 4a; Supplementary Table 6). Regarding plant architecture, 85, 83, and 81% of crosses should produce better progeny than Cavendish with 30 seeds for *Number of Leaves at Harvesting*, *Number of Leaves at Flowering*, and *Plant Girth,* respectively. Conversely, if the breeding target is *Robustness Index,* no cross should produce a progeny more robust than Cavendish with reasonable progeny size. For *Pseudostem Height*, the situation was intermediate in that 6% of crosses should produce a better progeny than Cavendish with 30 seeds. Regarding bunch and fruit architecture traits, the percentage of crosses producing such a progeny ranged respectively from 48 to 81% and 19 to 71% according to the trait. Finally, for yield component traits, 78% of crosses should produce a progeny with higher *Number of Fruits per Hand* and lower *Days to Fruit Maturity* than Cavendish with 30 seeds, dropping to less than 50% of the crosses for *Fruit Weight*, *Bunch Weight*, *Number of Fruits*, or *Number of Hands*.

With 30 seeds produced, one cross (Pisang Madu × PakaT) has the potential to produce progenies that could outperform Cavendish for each trait taken independently, except for *Robustness Index*, *Pseudostem Height* and *Fruit Pedicel Length* (Fig. 4b;  Supplementary Table 6). Note that this does not mean that 30 seeds are sufficient to obtain a progeny that could combine better performance than Cavendish for all these traits.

#### Genomic heritability

A 2x–4x hybrid genomic model was proposed, which incorporates genomic information through the genomic relationship of GCA and SCA terms. The SCA term was further split into dominance and across-population epistasis contributions. The significance of each random term was evaluated using likelihood-ratio(LR) tests applied to nested models taking into account genetic effects with gradual complexity (Supplementary Table 7). Most genetic effects were significant for all traits, except for the across-population epistasis contribution to the SCA effect that was significant for only ten traits, namely *Bunch Weight, Days to Fruit Maturity, Leaf Length, Number of Fruits, Number of leaves at Flowering, Peduncle Diameter, Peduncle Length, Pseudostem Girth, Pseudostem Height* and *Robustness Index*, when compared to a model accounting already for the contribution of dominance to the SCA effect. This comparison of models revealed issues in estimating the two SCA components with transfer effects between them (Supplementary Table 7 and Supplementary Fig. 4).

The contribution of dominance to the SCA effect included an expectation term related to the expected inbreeding of the cross. For all traits, the estimated inbreeding effects contributed very little to the overall variance and were significant only for *Leaf Width, Number of Hands,* and *Number of Fruits* (Supplementary Table 8). Interestingly, the genomic heritability of the complete genomic model (Supplementary Table 8) was higher than those obtained from the phenotypic model (Fig. 1a and Supplementary Table 3) for all traits except for *Pseudostem Girth*, *Fruit Length* and *Fruit Weight*.

#### Genomic prediction

**Fig. 5.**
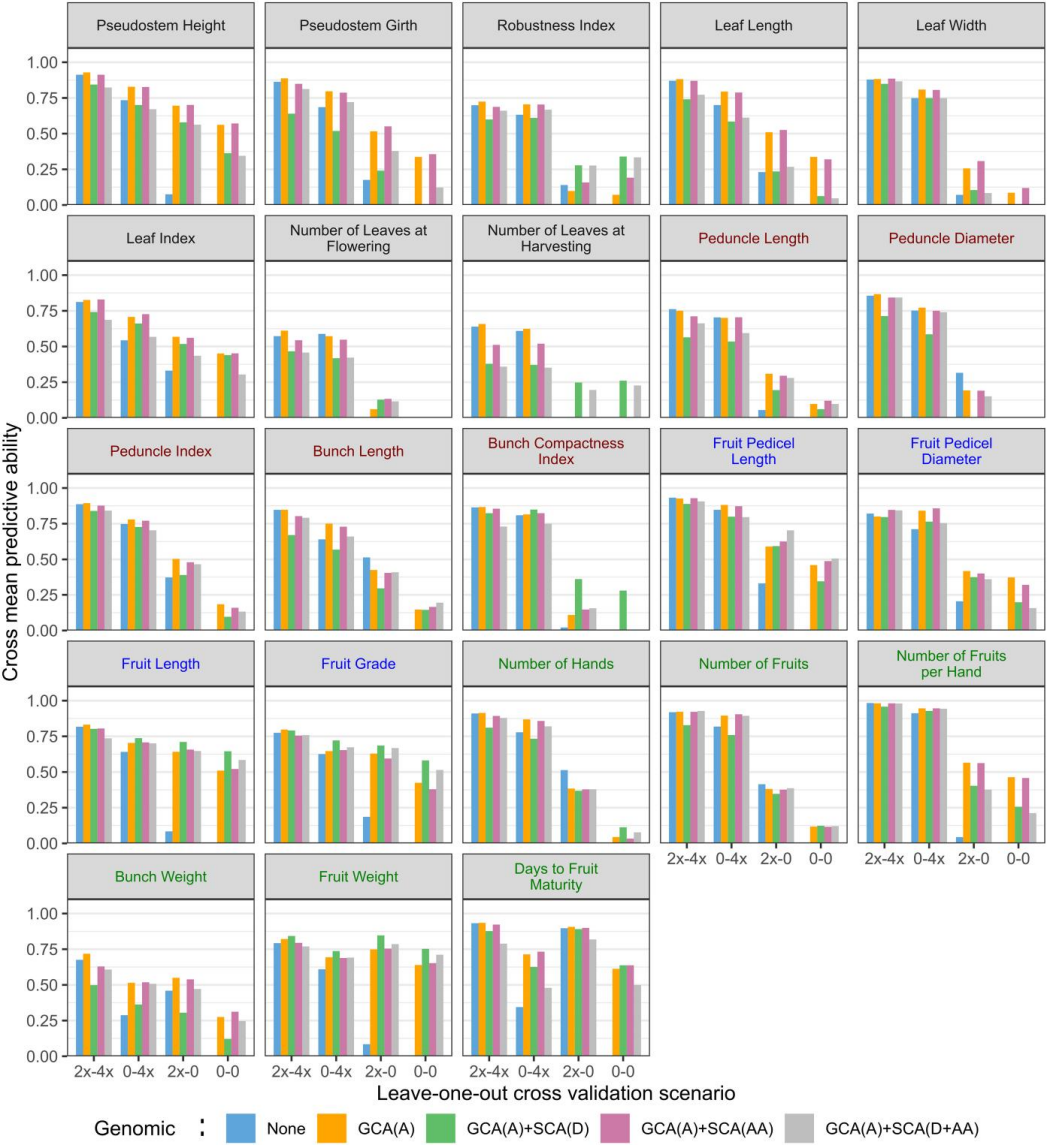
Cross mean predictive ability evaluated by leave-one-out cross-validation. Different models were compared, differing in the type of genetic effects incorporating genomic information: none “None,” in GCA terms only “GCA(A),” in GCA terms and in the dominance contribution to the SCA component “GCA(A) + SCA(D),” in the across-population epistasis contribution to the SCA component “GCA(A) + SCA(AA),” or both “GCA(A) + SCA(D + AA).” Traits related to plant stature, bunch architecture, fruit architecture, and yield components are colored in black, red, blue, and green, respectively. The *y*-axis is bounded between zero and one. Occasional negative values were observed (Supplementary Table 9), particularly under scenario 0–0 with the lowest training population size and for traits with low heritability due to a well-known artefact of leave-one-out cross-validation methodology (Zhou *et al*. 2016).

Different genomic prediction models were applied, differing in the type of genetic effects incorporating genomic information: none, in GCA terms only “GCA(A)”, in GCA terms and in the dominance contribution to the SCA component “GCA(A) + SCA(D)”, in the across-population epistasis contribution to the SCA component “GCA(A) + SCA(AA),” or both in the complete model “GCA(A) + SCA(D + AA).” These genomic models were evaluated for their predictive ability and compared to the phenotypic model (Fig. 5; Supplementary Table 9).

When both parents have already been observed in other crosses of the design (2x–4x scenario), the mean performance of a new cross is generally well predicted regardless of the model (*r* > 0.5). When one parent is missing, predictions tend to be more accurate when the 2x parent is unobserved (0–4x scenario) than when the 4x parent is unobserved (2x–0 scenario), except for *Bunch Weight*, *Fruit Weight*, and *Days to Fruit Maturity*. When none of the parents have been observed in other crosses (0–0 scenario), no prediction is possible without parent genotyping (Fig. 5).

The use of genomic relationship matrices in GCA and SCA terms to predict cross-mean performance has generally improved predictive abilities compared to the model using only phenotypic and pedigree information, especially in scenarios where at least one of the parents was not observed in another cross. The improvement in predictive ability was often more pronounced when the unobserved parent was the 4x parent (i.e. 2x–0 scenario). In the most difficult scenario where none of the parents have been observed in other crosses (i.e. 0–0 scenario), predictive abilities were null for some traits regardless of the model like *Peduncle Diameter*, but could exceed 0.5 for others like *Fruit Weight*.

The relative performance of genomic prediction models was variable according to the trait. For fruit traits like *Fruit Length*, *Fruit Grade,* and *Fruit Weight*, the modeling of dominance in SCA terms allowed to improve the predictive ability, e.g. *r* = 0.425 for “GCA(A)” and *r* = 0.581 for “GCA(A) + SCA(D)” for *Fruit Grade* in the 0–0 scenario. For other traits like *Pseudostem Height*, the predictive ability dropped from *r* = 0.562 for “GCA(A)” to *r* = 0.363 “GCA(A)+SCA(D)” in the 0–0 scenario. The modeling of across-population epistasis in SCA terms had a very limited impact with similar predictive abilities obtained between models differing only by this term, e.g. when comparing model “GCA(A)” vs model “GCA(A) + SCA(AA).”

## Discussion

### Evaluation of agro-morphological traits

The 23 traits related to yield components, as well as plant, bunch, and fruit architecture, used to evaluate hybrids in the first unreplicated experimental phase of CIRAD's breeding program showed moderate to high heritabilities, confirming the good evaluation of plant genotypic values.

Several groups of traits appeared as highly correlated and could be related to: plant biomass (*Pseudostem Height, Pseudostem Girth, Leaf Length, Leaf Width, Leaf Index*), bunch biomass (*Peduncle Diameter, Bunch Length, Bunch Weight, Number of Hands, Number of Fruits,* and *Number of Fruits per Hand*) and fruit biomass (*Pedicel Diameter, Fruit Length, Fruit Width,* and *Fruit Weight*). These observations agree with those of Nyine *et al*. (2017) in an East African banana genomic selection population.

From the perspective of yield, defined as the amount of bananas produced per unit of land area and time, some tradeoffs exist between key components. Notably, *Number of Fruits* per plant was negatively correlated with *Fruit Weight*. The growth rate of fruits depends on source-sink relationships after inflorescence emergence, specifically on the availability of carbon molecules (supply) and the number of fruit cells to be filled (sink demand) (Jullien *et al*. 2001). During fruit filling, the available carbon will be distributed among the different sinks (bananas), leading to this tradeoff between the number of fruits and their weight. This could also explain the positive correlation between bunch biomass components and *Number of Leaves at Harvesting* in our study. However, Rapetti and Dorel (2022) demonstrated that the active leaf area at floral induction/fruit number ratio varied among 12 Cavendish cultivars, suggesting that at least clonal variability in carbon allocation exists in bananas. In a genomic selection population of 307 genotypes, Nyine *et al*. (2017) observed that genotypes with poor fruit filling were not significantly influenced by cycle and field inputs. They hypothesized that differences in C-source to C-sink capacity may exist among banana genotypes. Non-susceptibility to source decreases could be an interesting breeding target for agroecological cropping systems. Breeding for such traits could be informed by mathematical models of plant growth that help design high-yield ideotypes. For instance, Qi *et al*. (2010) showed how optimizing plant growth model parameters could provide insights into the dynamics of source-sink relationships in crops like maize. The authors suggested breeding for optimal tradeoffs of source-sink dynamics instead of using the harvest index as an evaluation factor for yield improvement.

### Unbalanced contribution of parents to genetic variance

The crossing design of this study implies mainly *Musa acuminata* domesticated and wild accessions, with each parent either used in the diploid or tetraploid (double-diploid) state.

The model used to correct the phenotypic values of banana hybrids for spatial effects (phenotypic 2x–4x genetic model) accounts for GCA and SCA effects of the diploid and tetraploid parents, as well as within-cross effects. Similar models are commonly used in maize genetic studies, and more generally among crop species for which cultivars are derived from hybrid breeding schemes (Technow *et al*. 2014; Crain *et al*. 2021; Fonseca *et al*. 2021).

The estimated proportion of variance associated with 2x and 4x GCA revealed that the tetraploid parent predominantly influences the hybrid performance over the diploid parent for 21 of the 23 traits. It could be explained by the fact that each hybrid inherits two chromosome copies from the tetraploid parent and only one from the diploid parent. It is consistent with results on triploid banana hybrids obtained from 4x to 2x crosses showing that maternal tetraploid GCA explains most of the variation in *Plant Height* and *Leaf Number* (Tenkouano *et al*. 2012b). In that experiment, the 4x parents used were natural 4x obtained from 3x to 2x crosses. The higher 4x GCA compared to the 2x GCA is likely amplified when the tetraploid parent is a doubled diploid as in the CIRAD breeding program. Indeed, the parent tends to transmit its complete genetic material to the progeny, especially when preferential pairing of identical copies occurs during meiosis. The consequence is that the variation goes more into the 4x GCA component (all 2x gametes tend to be similar) than in the within-cross component. In this context, the choice of the 4x parent is crucial to triploid banana breeding. However, some traits showed a different profile: equal contribution for *Leaf Index* or a lower 4x GCA than 2x GCA for *Days to Fruit Maturity*. If a QTL with a strong effect is heterozygous in a single parent used only in the diploid state, only crosses involving it will show segregation at this QTL. The variation induced by this QTL will be distributed in the 2x GCA components and in the within-cross component, but not in the 4x GCA component. This phenomenon could compensate for the lower number of copies transmitted by the diploid parent. Consistently with our results, 2x GCA effects were more important than tetraploid effects for *Days to Fruit Maturity* in studies involving plantain-derived tetraploid and wild diploid accession (Tenkouano *et al*. 2012a, 2012b).

The SCA contribution to overall genetic variance was low suggesting that, for most traits, additive genetic effects contribute more to genetic variance than non-additive genetic effects. The limited contribution of the SCA component to the genetic variance is often observed in hybrid crops (Joshi *et al*. 2004; Muraya *et al*. 2006; Khalil and Hatem 2014). As a result, the GCA of both parents is a good predictor of the cross-mean value.

One of the particularities of our study is that the hybrids evaluated were not F1 hybrids resulting from crosses between pure lines. They were hybrids between heterozygous parents, which produced non-identical gametes that vary across heterozygous loci. As a result, an additional variance component that accounts for within-cross variability must be taken into account (Ortiz 1997). In our study, this component was generally large and was the main contributor to the heritability for nine traits namely *Robustness Index, Leaf Index, Number of Leaves at Harvesting, Peduncle Length, Peduncle Index, Bunch Compactness Index, Fruit Pedicel Diameter, Fruit Grade*, and *Days to Fruit Maturity*. Therefore, the cross-mean value is not the only parameter that should be used to decide on the interest of the cross, but also its variance. However, inference of variance must be considered with caution. As each hybrid was only evaluated once, hybrid effects may be partially confounded by plot error, although replication of the checks and repeated measurements across cycles should mitigate this confounding of effects.

### Comparative performance of families

Families can be assessed based on their cross-mean performances using the two major principal components that account for yield, plant and bunch biomass for the first principal component and fruit biomass for the second principal component. Many crosses gathered around Cavendish are used as controls in the design, which makes sense as Cavendish has been the targeted ideotype of the breeding program for many years. Clustering of families is mainly driven by the 4x parent rather than the 2x parent. The clustering of 4x Mchare parent crosses around Cavendish was consistent with Mchare being the 2x donor of Cavendish (Martin *et al*. 2023). Compared to Calcutta 4, a wild cultivar, families showed on average better agronomic performances with bigger, longer, heavier fruits and a higher yield.

Families can also be assessed through the variability generated from each cross, which revealed large differences according to the cross, or by combining both the mean and the variance. Hence, crossbreeding selection can incorporate the genetic variance of descendants using various criteria known as CSCs (cross-selection criteria). The usefulness criterion (UC) developed by Schnell and Utz (1975) evaluates the expected performance of the best progenies from a cross. However, this criterion does not directly measure a cross's ability to produce exceptional offspring. To address this issue, Bijma *et al*. (2020) and Wellmann (2019) suggested quantifying a cross's utility by the probability that a descendant outperforms a certain threshold, assuming Gaussian assumptions. We have adopted this approach by using this probability to estimate the minimum progeny size to be generated in order to obtain a descendant exceeding a threshold value. For each trait, Cavendish performance was used as the threshold value, as it is the most traded variety (57% of global banana production) (Lescot 2023) and can thus be considered as a major ideotype of dessert bananas.

The calculation of minimum progeny size to be generated to have a descendant with better performance than Cavendish suggested very promising crosses except for *Plant Robustness* and *Plant Height*. One cross (Pisang Madu × PakaT) has the potential to produce, with a relatively low number of individuals, progenies that could outperform Cavendish for each trait taken independently, except for *Robustness Index*, *Pseudostem Height*, and *Fruit Pedicel Length*. The calculation of the minimum progeny size to be generated to have a descendant that combines those traits is more complex as it should be adjusted to account for genetic correlations between traits.

*Plant Robustness* and *Plant Height* are key traits to prevent lodging, but none of the crosses seem to have the ability to generate a progeny with comparable performance to Cavendish. These two traits have high heritability and good predictive ability, suggesting that they are strongly influenced by genetic factors. However, the variance observed for these traits is insufficient to produce a banana tree as modest in height (and therefore as robust) as Cavendish. This Cavendish cultivar, widely grown in the world, is believed to be a shorter somaclonal variant of an original tall Cavendish line (Simmonds 1954). Therefore, the exploitation of somaclonal variation may serve as a valuable complementary approach to conventional crossbreeding for the improvement of these traits.

### Accounting for parental genomic information in cross-mean prediction

In this study, we investigated the ability to predict the cross-mean performance using the genomic information of the parents, which was used to calculate genomic relationship matrices of GCA and SCA components. Based on the work of Endelman (2023, 2025), we split the SCA into two components: the contribution of dominance and across-population epistasis to the SCA effect (here populations correspond to 2x and 4x parental populations). The cross-validation scenarios 2x–4x, 0–4x, 2x–0, and 0–0 were adapted from the t0, t1, and t2 scenarios established within the maize hybrid prediction framework (Technow *et al*. 2014; Kadam *et al*. 2016; Seye *et al*. 2020). The distinction lies in delineating cases where only one parent is observed, based on the parental ploidy level. The estimates obtained from the genomic 2x–4x genetic model were generally consistent with those from the phenotypic 2x–4x model, although substantial gains in heritability were observed for certain traits when adding the genomic information of the parents (e.g. *Fruit Pedicel Diameter*). The genomic model also highlighted that both SCA components (dominance and across-population epistasis) contributed to most traits. When this was not the case, it likely resulted from an estimation issue generating a transfer phenomenon between these two components (e.g. *Fruit Length*, *Fruit Grade*, *Bunch Weight*, and *Fruit Weight*) (Supplementary Table 7 and Supplementary Fig. 4).

Predicting new parental combinations was highly accurate when both parents were already observed in the design. In this case, the genomic information of the parents contributed little or no additional gain. It could be explained by the fact that the GCA of both parents was accurately estimated on the basis of phenotypic information only. A significant asymmetry was observed between the 0–4x and 2x–0 scenarios: predicting the mean performance of a cross, when the tetraploid parent is observed, is much easier than the opposite. This is consistent with the partitioning of variance between the 2x and 4x GCA components for most traits. In the 0–0 scenario where none of the parents have been observed in the design, only genomic prediction models could be used to obtain a prediction of cross-mean performance. The predictive abilities obtained were very variable according to the trait, ranging from null for *Peduncle Diameter* to values exceeding 0.65 for *Fruit Weight*, regardless of the model. The ability to predict cross-mean performance in the 0–0 scenario could be explained by the sharing of genetic information among crosses with related parents. The high variability in predictive abilities obtained according to the trait did not simply result from differences in heritability and needs to be examined more deeply to find its cause.

The benefits of taking genomic information into account through the two SCA components were very variable according to the trait, with substantial gains in predictive ability for fruit traits, but significant losses for other traits. These variable performances could be attributed to the difficulty in accurately estimating dominance and across-group epistasis, as already highlighted by González-Diéguez *et al*. (2021). We hypothesize that non-additive effects would benefit from genomic information at the hybrid level, rather than at the parental level.

These results are very promising regarding the ability to predict the mean performance of new crosses using genomics. However, as the genomic predictive ability determines the stringency with which crosses can be selected, only highly predictable traits like *Fruit Weight* can be prioritized with high confidence. In the future, it would be interesting to attempt to predict the variance of crosses using genomics, which would make it possible to identify the crosses most likely to generate promising hybrids. This would require the generalization to polyploid species of the theoretical work of Wolfe *et al*. (2021) on the prediction of cross variance in outbred species.

### Impact on Breeding Program Efficiency

The phenotyping and experimentation strategy used for the early selection phase proved effective, allowing moderate to high heritability to be obtained for the agro-morphological traits assessed. The traits associated with fruit size, such as *Fruit Length*, *Diameter*, and *Weight*, demonstrated high heritability (*H*² > 0.82) and predictive ability (ranging from 0.51 for fruit grade to 0.71 for *Fruit Weight*). Interestingly, these traits are of significant importance for the Cavendish export ideotype, suggesting highly promising results in the future for the breeding program.

Significant correlations were observed within and between trait categories. Hence, selection could be done for traits showing the highest heritability and that are easily measured enabling a gain of cost and time in the early selection phase. For example, *Bunch Weight* is a challenging trait to measure, with moderate heritability (0.41) and variable prediction accuracy (ranging from 0.3 to 0.7). Due to its significant positive correlation with *Fruit Weight*, the latter could replace it in early selection, as it is easier to measure, has much higher heritability (0.83), and exhibits stable prediction accuracy (ranging from 0.7 to 0.8). Similarly, we could implement early selection based on the *Number of Fruits* rather than *Peduncle Diameter*, *Leaf Length*, *Leaf Width*, *Fruit Pedicel Length*, and *Fruit Pedicel Diameter*. Indeed, these latter traits are significantly correlated with *Number of Fruits* and are breeding targets with a broad range of acceptable values.

Our results highlighted the importance of choosing carefully the tetraploid (4x) parent, suggesting the need to increase the number of potential parents used in 4x, or even to directly improve a population of 4x parents to enhance genetic gain. Promising parental combinations have been identified, in particular one likely to outperform the Cavendish ideotype, justifying increased efforts on this cross and on those involving similar genetic backgrounds. This strategy could be extended to other ideotypes beyond the Cavendish dessert banana and to other traits like fruit quality and disease susceptibility.

The banana breeding program can be seen as a reciprocal recurrent selection breeding program involving two main components: (i) the improvement of parental populations and (ii) the identification of superior triploid hybrids to be registered as cultivars. For the first component, genomic prediction could be applied through parental genotyping, a cost-effective method that requires genotyping a relatively small number of parents. It allows the identification of parents with high breeding values based on predicted GCA, and promising 2x–4x combinations, possibly based on predicted SCA. For the second component of the breeding program, genomic prediction could be applied through hybrid genotyping, a more expensive approach that requires genotyping a larger set of individuals, typically a few thousand hybrids in the early phases of the program. Here, the goal is to predict the total hybrid genotypic value in order to streamline phenotyping efforts by focusing on the most promising hybrids. While this second application remains to be evaluated, interesting predictive abilities (0.47–0.75) were obtained in this context by Nyine *et al*. (2018) for fruit filling and fruit bunch traits. Overall, these results along with those from our study show the potential of genomic prediction to improve banana breeding.

## Supplementary Material

iyaf119_Supplementary_Data

## Data Availability

All phenotypic and genotypic data underlying this study are available from the following CIRAD Dataverse repository: https://doi.org/10.18167/DVN1/YSM8R7, including the vcf file “ParentVcf_filt.vcf.gz”, the pedigree information and raw phenotypic data for all traits in “rawPhenotyping_2723hybrids_23traits.csv”. Raw GBS data are available in the SRA database (PRJNA1252056). Supplemental material available at *GENETICS* online.
