# Supplementary material for: Combining ability of banana triploid hybrid progenitors and genomic prediction of cross performance for agro-morphological traits
Source: Genetics. 2025 Jun 20;230(4):iyaf119. doi: 10.1093/genetics/iyaf119 (PMC12341918; doi:10.1093/genetics/iyaf119)
Supplement: iyaf119_Supplementary_Data [file iyaf119_supplementary_data.zip › Supplementary_Figures_and_Legends_GENETICS-2025-308015.docx]

**SUPPLEMENTARY INFORMATION**

Combining ability of banana triploid hybrid progenitors and genomic prediction of cross performance for agro-morphological traits

L. Toniutti^2,3,α^, S. Rio^1,2,α^, C. Madec^2,3^, S. Ricci^1,2^, C. Guiougou^2,3^, F. Marius^2,3^, C. Mina^2,3^, J.M. Delos^2,3^ , F. Lambert^2,3^, , J.C. Efile^2,3^, A D’Hont^1,2^, G. Martin^2,3β^, J.Y. Hoarau^2,4,β^,F. Salmon^2,3β^

**TABLE OF CONTENT:**

**SUPPEMENTARY FIGURES:**

Figure S1: Experimental design A. Example of 2 plots : Plot 1 is composed of 2 blocks whereas plot 2 is composed of 8 blocks. B. Zoom in one block : One block contains 56 hybrids and 8 control plants ( 5 Cavendish, 1 Pisang Ceylan, 1 Pisang Madu and 1 calcutta 4)

Figure S2: Schematic representation of the four Leave-One-Out cross-validation scenarios (represented as rows): 2x-4x, 2x-0, 0-4x, and 0-0. Each cross (represented as columns) is predicted by discarding cross data of the training set according to the cross-validation scenario

Figure S3: Number of hybrids included to fit the model in each Leave-One-Out cross-validation scenario

Figure S4: Heritability partitioning using genomic information

**SUPPLEMENTARY TABLES** (see associated SUPPLEMENTARY_TABLES_S1-S5.xlsx file):

**Table S1:** Number of offspring for all crosses present in the experiment

**Table S2:** Data used for genotyping of parents

**Table S3:** Model parameters and heritability of the phenotypic model

**Table S4:** Likelihood ratio tests of nested phenotypic models including: a null model with no random genetic term (model 0), a model with the hybrid genetic component only (model 1), a model with 2x and 4x GCA components with equal variances (model 2), or specific variances (model 3), and the complete model with the SCA component (model 4)

**Table S5:** Pearson’s correlation coefficients of traits

**Table S6:** Progeny size to be generated to have an offspring with a better performance than Cavendish for 20 traits

**Table S7:** Likelihood ratio tests of nested genomic models including: a null model with no random genetic term (model 0), a model with the hybrid genetic component only (model 1), a model with 2x and 4x GCA components with equal variances (model 2), or specific variances (model 3), a model with the dominance contribution to the SCA effect (model 4), and the complete model with the across-population epistasis contribution to the SCA effect (model 5)

**Table S8:** Model parameters and heritability of the genomic model

**Table S9:** Cross mean predictive abilities evaluated by leave-one-out cross validation

**SUPPEMENTARY FIGURES:**


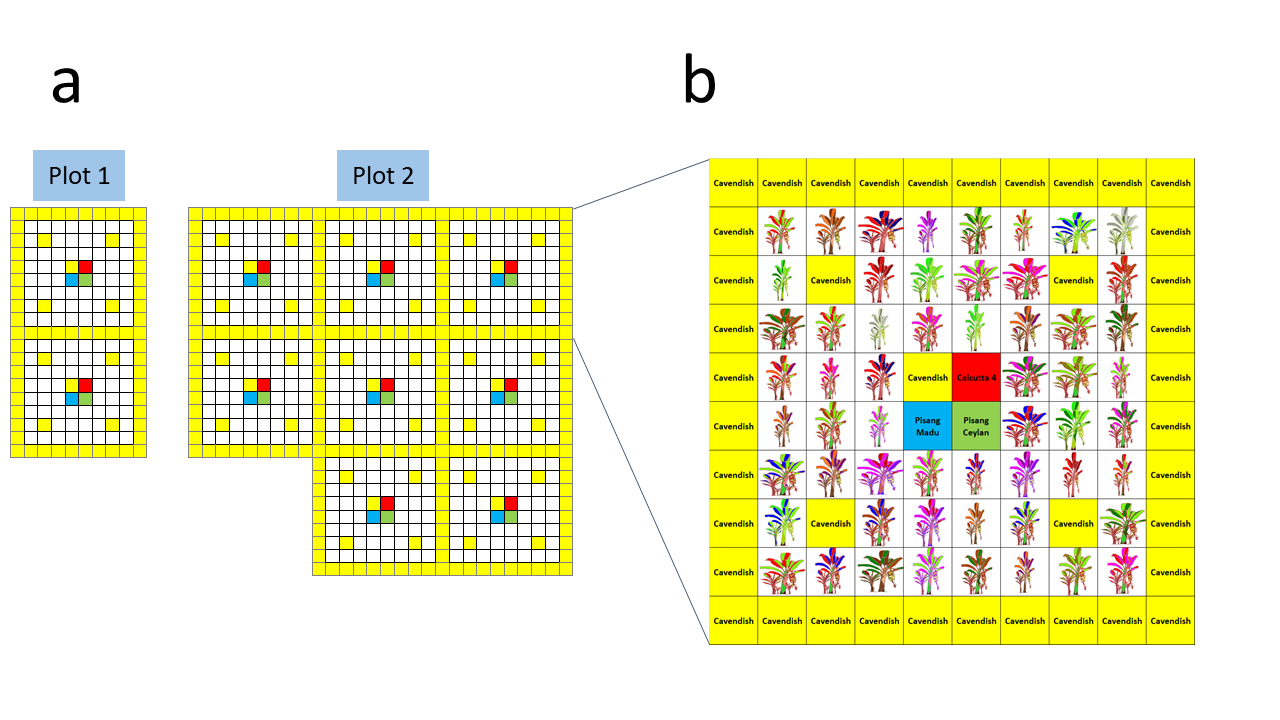


**Figure S1: Experimental design** a. Example of two plots: Plot 1 is composed of two blocks whereas Plot 2 is composed of eight blocks. b. Zoom in on one block: one block contains 56 hybrids and eight control plants (five Cavendish, one Pisang Ceylan, one Pisang Madu and one Calcutta 4)


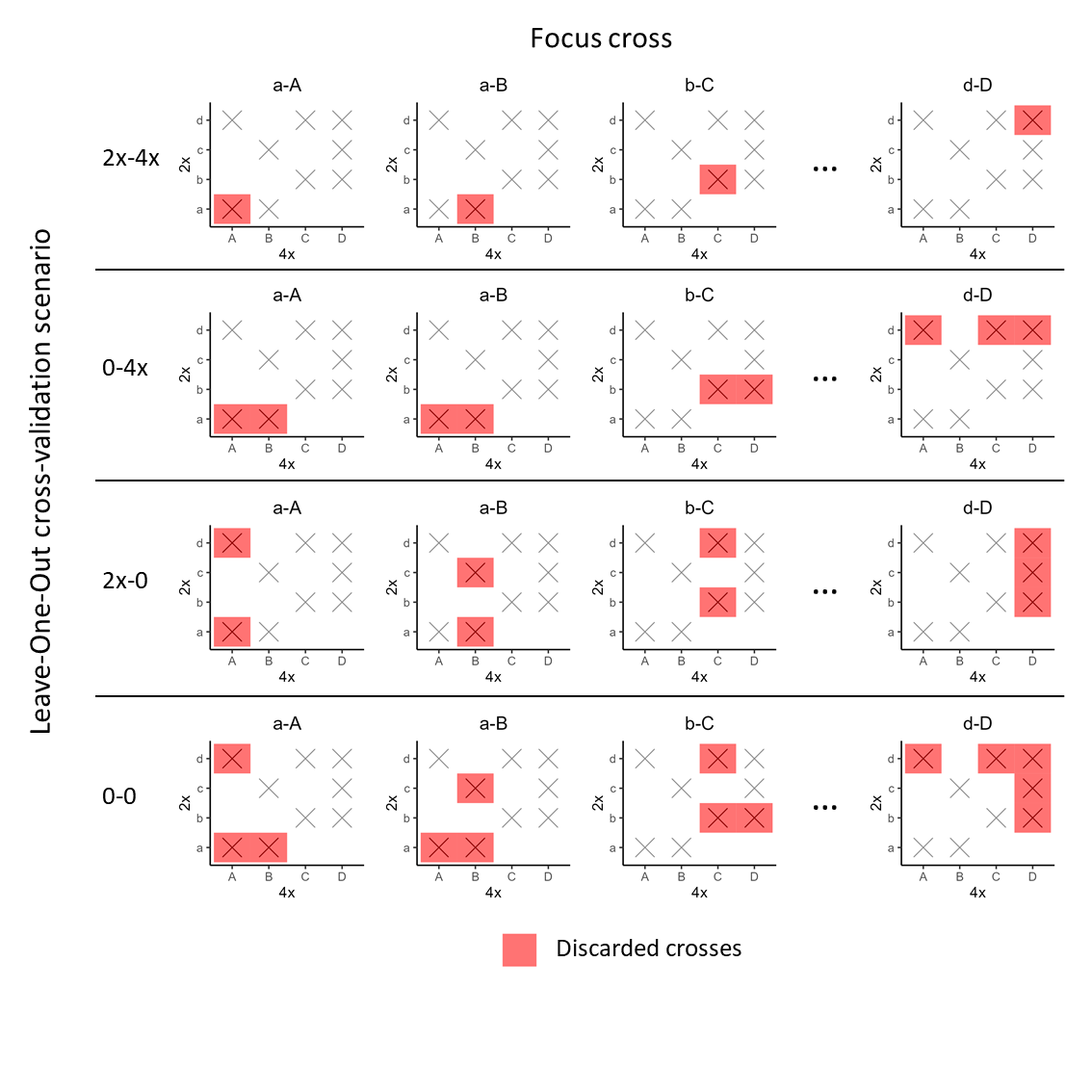


**Figure S2: Schematic representation of the four Leave-One-Out cross-validation scenarios (represented as rows): 2x-4x, 2x-0, 0-4x, and 0-0.** Each cross (represented as columns) is predicted by discarding cross data of the training set according to the cross-validation scenario

**
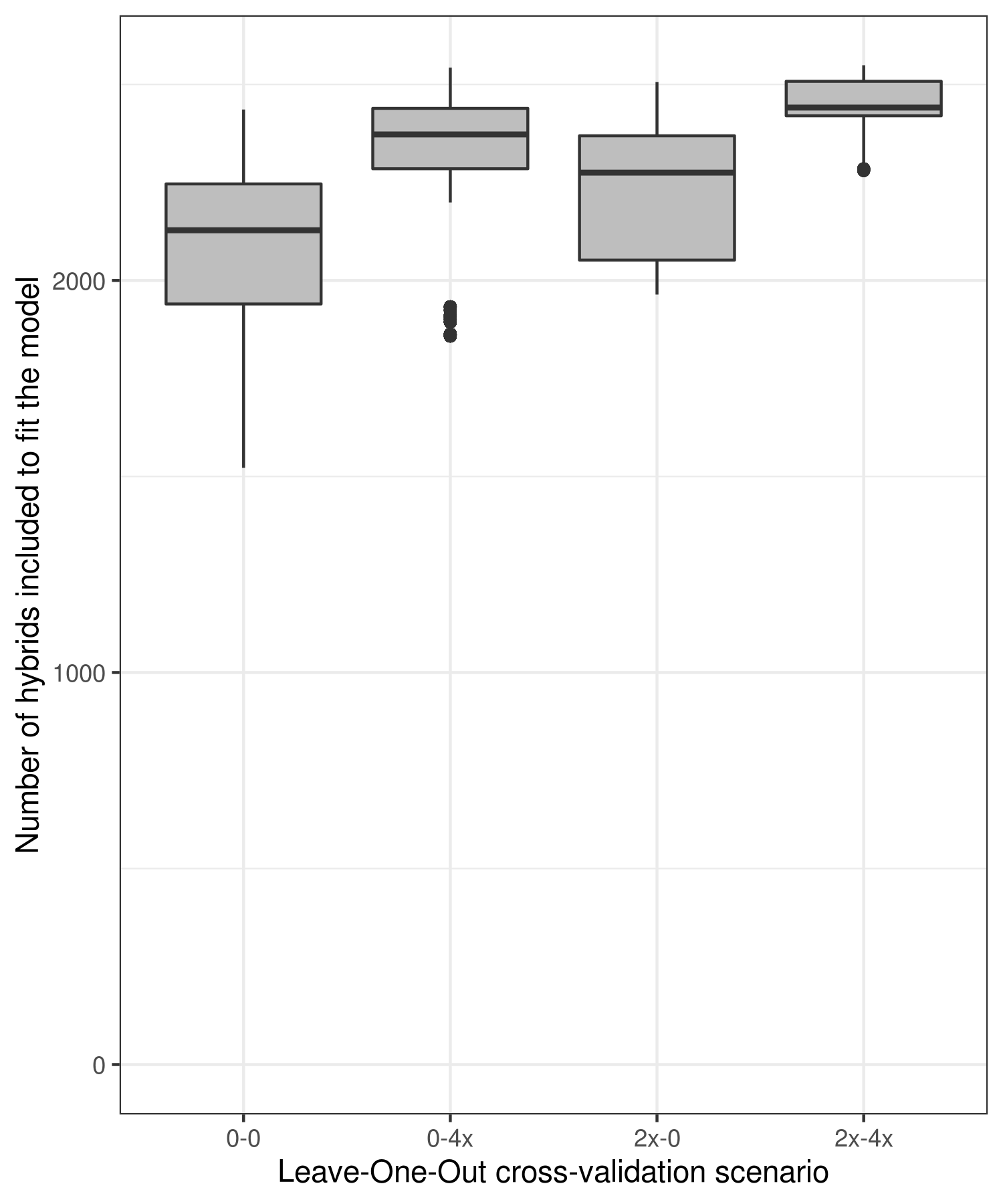
**

**Figure S3: Number of hybrids included to fit the model in each Leave-One-Out cross-validation scenario**


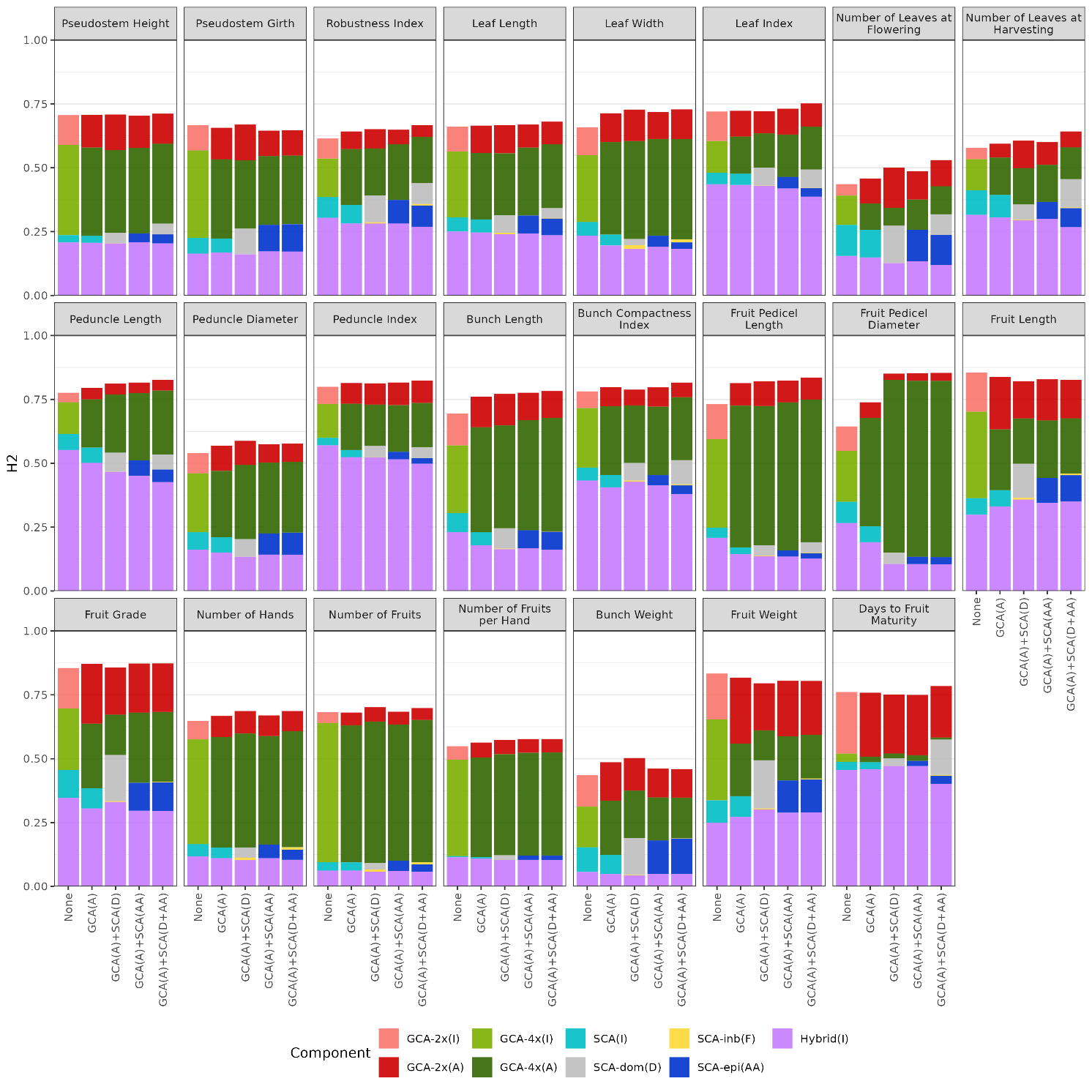


**Figure S4: Heritability partitioning using genomic information.** Different models were compared, differing in the type of genetic effects incorporating genomic information: none “None”, in GCA terms only “GCA(A)”, in GCA terms and in the dominance contribution to the SCA component “GCA(A)+SCA(D)”, in the across-population epistasis contribution to the SCA component “GCA(A)+SCA(AA)”, or both “GCA(A)+SCA(D+AA)”. Each component is indicated with a specific color and the incidence and covariance matrices are indicated between brackets for fixed and random terms, respectively: F corresponds to the expected inbreeding coefficient, I to the identity matrix, A to the additive genomic relationship matrix, D to the expected dominance genomic relationship matrix, AA to the Kronecker product between 2x and 4x additive genomic relationship matrices
